# A Novel Homozygous Truncating *CD8A* Variant (p.Arg107Ter) in a Patient with Recurrent Sinopulmonary Infections: A Case Report and Literature Review

**DOI:** 10.3390/healthcare14070969

**Published:** 2026-04-07

**Authors:** Ali A. Asseri, Ebtesam Elgezawy, Sarah Ibrahim Summan, Abdullah A. Alamoudi, Ashwag Asiri

**Affiliations:** 1 Department of Child Health, King Khalid University, Abha 62529, Saudi Arabia; asalasiri@kku.edu.sa; 2 Department of Immunology, Abha Maternity and Children Hospital, Ministry of Health, Abha 62521, Saudi Arabia; eelgezawy1@yahoo.com; 3 Department of Pediatrics, Abha Maternity and Children Hospital, Ministry of Health, Abha 62521, Saudi Arabia; saraibraheem@hotmail.com; 4 Department of Pediatric Gastroenterology, King Khalid University Medical City, Abha 62529, Saudi Arabia; aalamodi@kku.edu.sa

**Keywords:** sinopulmonary infection, children, CD8α deficiency, combined immunodeficiency

## Abstract

**Background**: *CD8A*-related CD8α deficiency (Immunodeficiency 116) is a rare autosomal recessive primary immunodeficiency disease characterized by absent CD8^+^ T cells and variable sinopulmonary disease. **Case Presentation**: A seven-year-old boy from a consanguineous family was referred for chronic wet cough and “uncontrolled asthma” despite being prescribed high-dose inhaled corticosteroids and montelukast. He was hospitalized seven times over a two-year period for presumed asthma exacerbations complicated by pneumonia. An examination revealed bilateral crackles without wheezing. Throat culture tested positive for *Haemophilus influenzae*. CT imaging showed signs of chronic rhinosinusitis (maxillary mucosal thickening) and chronic airway disease with bronchiectatic changes. The patient’s immunoglobulin levels were within normal ranges for his age group. Flow cytometry revealed profound CD8^+^ T-cell lymphopenia (CD8^+^ 0.21%; 11 cells/µL; near-absent after excluding dual-positive cells) with expansion of CD3^+^CD4^−^CD8^−^ T cells (29.5%). *CD8A* gene sequencing identified a novel homozygous nonsense variant NM_001768.7:c.319C>T (p.Arg107Ter; GRCh38: chr2:86790412G>A), consistent with loss of CD8α and secondary loss of CD8β surface expression. A literature review identified three previously reported symptomatic patients (and two asymptomatic sisters in the first family), all with recurrent respiratory infections and variable structural lung disease. **Conclusions**: This case highlights *CD8A* deficiency as a rare mimic of pediatric asthma and expands the genotype spectrum with a truncating *CD8A* variant. Early lymphocyte immunophenotyping in children with recurrent sinopulmonary infections may prevent delayed diagnosis and progressive airway damage.

## 1. Introduction

CD8 functions as an essential coreceptor that strengthens the T-cell receptor (TCR) recognition of peptides presented by major histocompatibility complex (MHC) class I molecules, thereby supporting the activation and effector function of cytotoxic αβ T lymphocytes [1,2]. Through its interactions with β2-microglobulin and the α2/α3 domains of MHC class I, CD8 enhances immune synapse stability and facilitates target-cell killing, contributing to host defense against infected, malignant, and allogeneic cells. On the cell surface, CD8 exists as either an αα homodimer or an αβ heterodimer; importantly, stable surface expression of CD8β depends on the presence of CD8α. When CD8α is absent or dysfunctional, CD8β is typically retained intracellularly and degraded, resulting in a marked reduction in or absence of CD8^+^ T cells [1,2,3,4,5].

Pathogenic variation in *CD8A*, the gene encoding the CD8α chain, causes an exceptionally rare form of immunodeficiency. CD8α deficiency (Immunodeficiency 116, OMIM #608957) is a rare autosomal recessive primary immunodeficiency (PID) disease caused by homozygous mutations in the *CD8A* gene that result in a complete absence of CD8^+^ T cells [2]. To date, only three clinically affected patients with genetically confirmed *CD8A*-related CD8α deficiency have been documented in the medical literature [2,4,5]. Notably, the first reported family also included two sisters with the same immunophenotype who remained asymptomatic, highlighting considerable clinical variability. Across reported symptomatic individuals, the phenotype ranges from recurrent sinopulmonary infections with progressive airway disease to milder respiratory involvement [5]. In this report, we describe a patient with a novel homozygous truncating *CD8A* variant (p.Arg107Ter) presenting with recurrent sinopulmonary infections, and we review previously published cases to further explore the clinical and genetic spectrum of *CD8A* deficiency.

## 2. Literature Review

We performed a systematic search across four electronic databases—PubMed, Ovid MEDLINE, ScienceDirect, and Web of Science—up to January 2026. The search strategy was designed to identify publications describing *CD8A*/CD8α deficiency, including genetic, clinical, and immunological characterizations. Search terms were combined using Boolean operators and included the following: (“CD8A” OR “CD8 alpha” OR “CD8α” OR “CD8 deficiency” OR “CD8 immunodeficiency”) AND (“mutation” OR “variant” OR “pathogenic” OR “loss of function” OR “truncating” OR “homozygous”) AND (“recurrent infections” OR “sinopulmonary” OR “bronchiectasis” OR “respiratory infections” OR “combined immunodeficiency”). Published cases, including the present case, are summarized in Table 1. 

## 3. Case Description

A seven-year-old boy was referred to the pediatric pulmonology clinic at Abha Maternity and Children’s Hospital for evaluation of chronic wet cough and uncontrolled asthma. The child was first diagnosed with asthma at age five and had since experienced frequent exacerbations, often triggered by upper respiratory tract infections and physical activity. Despite adherence to high-dose inhaled corticosteroids and montelukast, his symptoms persisted. Over the preceding two years, the patient had been hospitalized seven times for presumed asthma exacerbations complicated by pneumonia, requiring intravenous antibiotics and systemic corticosteroids. Notably, there was no history of tuberculosis exposure, chronic diarrhea, or significant neurological or genitourinary symptoms. Family history revealed consanguinity and a paternal history of asthma but no known immunodeficiencies.

On examination, the child appeared well and was breathing comfortably. Vital signs were stable, with a respiratory rate of 25/min and oxygen saturation of 95% on room air. His growth parameters were within the 25th percentile for weight and height. Chest auscultation revealed bilateral crackles without wheezing. No clubbing, cyanosis, or dysmorphic features were noted.

The patient’s hematological, biochemical, and microbiological findings were within normal reference ranges. He had a white blood cell count of 11 × 10^9^/L (within normal limits), hemoglobin of 12.5 g/dL, and platelet count of 342 × 10^9^/L. His inflammatory markers showed an elevated ESR (34 mm/h) with normal CRP (3 mg/L). The results of the liver and renal function tests were normal. A throat swab culture revealed a strong presence of *Haemophilus influenzae*. The patient’s immunoglobulin levels were within normal ranges, and sputum testing for acid-fast bacilli was negative.

**Figure 1:**
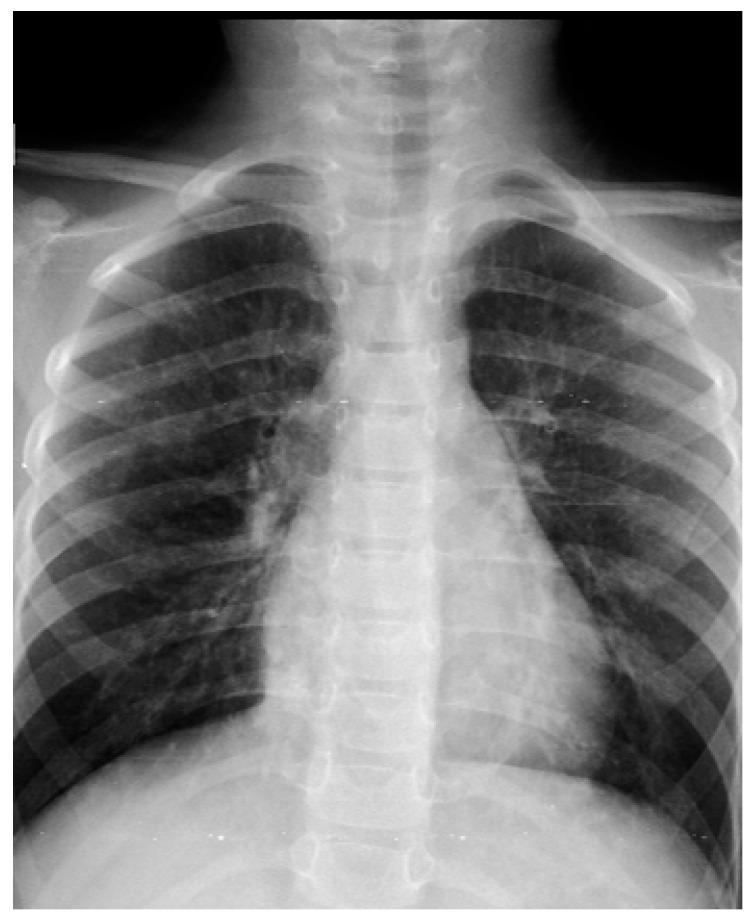
Plain chest radiography demonstrated bilateral perihilar peribronchial thickening with mild hyperinflation, without focal lobar consolidation or pleural effusion—findings consistent with chronic/recurrent airway inflammation.

**Figure 2:**
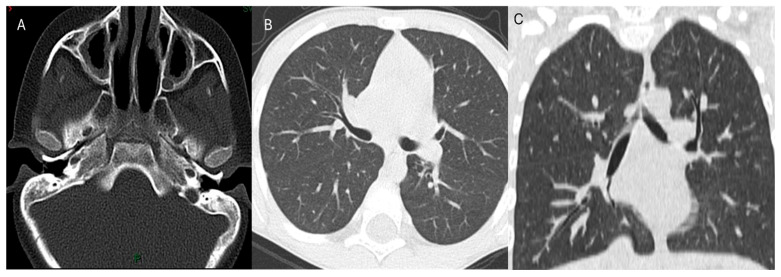
(**A**) Paranasal sinus CT: Axial CT demonstrates bilateral maxillary sinus mucosal thickening without a definite air–fluid level or bony erosion, consistent with chronic rhinosinusitis. (**B**,**C**) Chest CT (axial and coronal lung windows) shows no focal consolidation or pleural effusion. Coronal images show chronic airway changes with cylindrical bronchiectasis.

**Figure 3:**
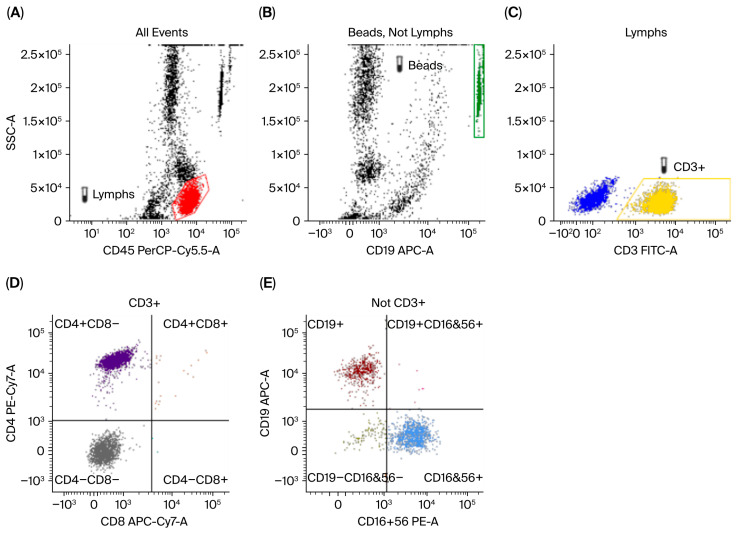
A flow-cytometric immunophenotyping and gating strategy demonstrating marked reduction in CD8^+^ T cells. (**A**) From all acquired events, lymphocytes were identified by gating the CD45^bright/SSC^low population (“Lymphs”) on CD45 PerCP-Cy5.5 vs. SSC-A. (**B**) Counting beads were identified as a discrete high-signal population (“Beads”) on CD19 APC vs. SSC-A and excluded from cellular analyses (or used for absolute counting, as applicable). (**C**) Within the lymphocyte gate, CD3^+^ T lymphocytes were defined by CD3 FITC expression and selected for downstream subset analysis. (**D**) Among CD3^+^ events, CD4 PE-Cy7 vs. CD8 APC-Cy7 quadrant gating revealed a predominance of CD4^+^CD8^−^ cells with markedly reduced/near-absent CD8^+^ events, resulting in a relatively increased CD4^−^CD8^−^ (double-negative) CD3^+^ compartment compared with expected distributions. (**E**) Within the CD3^−^ fraction, B cells and NK cells were identified using CD19 APC vs. CD16+56 PE, defining CD19^+^CD16/56^−^ (B cells) and CD19^−^CD16/56^+^ (NK cells); the CD19^−^CD16/56^−^ population is shown for completeness. Axes are displayed on logarithmic scales for fluorescence parameters.

Imaging studies, including chest X-ray and high-resolution CT, demonstrated features of chronic lung disease and sinusitis (Figure 1 and Figure 2). Lymphocyte subset analysis revealed a marked predominance of T cells (CD3^+^), accounting for 73.6% of lymphocytes (3758 cells/µL), with CD4^+^ T cells comprising 44.1% (2254 cells/µL). In contrast, CD8^+^ T cells were profoundly reduced at only 0.21% (11 cells/µL), and after dual-positive cells were excluded, they were almost completely absent (0.02%, 1 cell/µL). A substantial proportion of double-negative T cells (CD3^+^CD4^−^CD8^−^) was observed, reaching 29.5% (1503 cells/µL). B cells (CD19^+^) represented 7.9% (403 cells/µL) of the total, while NK cells (CD3^−^CD16^+^CD56^+^) accounted for 17.0% (870 cells/µL). These findings indicate severe CD8^+^ T-cell deficiency with compensatory expansion of double-negative T cells, consistent with CD8α deficiency (Figure 3). CD8 expression was assessed by flow cytometry using the anti-CD8 monoclonal antibody clone SK1, in accordance with our local laboratory protocol. This clone binds an epitope located within the extracellular domain of the CD8α chain.

In light of the patient’s profound CD8^+^ T-cell deficiency, we sequenced the *CD8A* gene encoding the CD8α chain. This gene is associated with autosomal-recessive Immunodeficiency 116 (OMIM: 608957). Genetic testing was performed using whole-exome sequencing (WES) on genomic DNA extracted from a dried blood spot. The exome was captured using the xGen Exome Research Panel v2 with supplemental mitochondrial and custom panels, and sequencing was conducted on the Illumina NovaSeq X system. Over 11.2 billion bases were generated with a high average coverage of 155×, covering 99.6% of targeted regions at ≥20×. Variants—including SNVs, INDELs, CNVs, mitochondrial variants, repeat expansions, mobile element insertions, and regions of homozygosity—were detected using a comprehensive bioinformatics pipeline (GATK v4.4.0.0, Manta v1.6.0, 3bCNV v2.1, Mutect2 v4.4.0.0, ExpansionHunter v5.0.0, MELT v2.2.2, and AutoMap v1.2) and annotated with Ensembl VEP. Interpretation followed ACMG/AMP guidelines, and only clinically relevant variants were reported [6].

**Figure 4:**
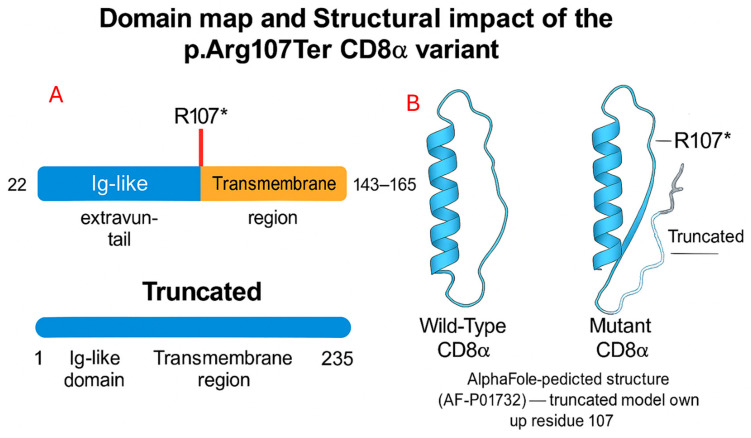
(**A**) Comparison of the full-length and truncated *CD8A* protein highlighting the position of the p.Arg107Ter variant. The nonsense mutation (red line) introduces a premature stop codon at residue 107, predicting loss of the downstream transmembrane and cytoplasmic domains and resulting in loss of normal membrane localization of the *CD8A* protein. (**B**) Structural comparison of wild-type and mutant CD8α. The wild-type CD8α displays an intact Ig-like domain, whereas the p.Arg107Ter mutation produces a truncated protein lacking downstream structural elements, indicating loss of normal folding and function. Note: This figure was partially generated using Microsoft Copilot (version M15.1) as an illustration-assistance tool, with all scientific content, annotations, and accuracy manually reviewed and validated by the authors.

Our analysis identified a homozygous, likely pathogenic exonic variant (NM_001768.7:c.319C>T) at genomic position 2-86790412-G-A (GRCh38) that results in a premature stop codon (NP_001759.3:p.Arg107Ter). This variant has not been previously reported in other patients. Unlike the previously described missense mutation (c.331G>A; p.Gly111Ser) affecting the immunoglobulin domain of CD8α [4], our patient’s nonsense mutation is predicted to cause complete loss of CD8α function, supporting the diagnosis of CD8α deficiency (Figure 4).

The patient was diagnosed with chronic suppurative lung disease (CSLD) secondary to CD8α deficiency. Asthma medications were gradually tapered, and the patient was initiated on prophylactic antibiotics and regular airway clearance. He was enrolled in regular follow-up monitoring for CSLD and chronic sinusitis.

## 4. Discussion

Our report describes a 7-year-old boy with CD8α deficiency caused by a homozygous truncating *CD8A* variant (p.Arg107Ter), extending the clinical and genetic spectrum of this exceptionally rare but clinically significant PID that can mimic common pediatric respiratory conditions such as asthma. This case highlights the diagnostic challenges in children initially labeled as having asthma, as asthma remains a clinical diagnosis and some patients are retrospectively identified only after showing response to therapy. Red flags should prompt consideration of alternative diagnoses, including immunodeficiency, include persistent wet cough, recurrent bacterial bronchitis, chronic sinusitis, and failure to improve after 4–6 weeks of appropriate asthma treatment. The *CD8A* gene encodes the α chain of the CD8 molecule, which is essential for the development and function of cytotoxic T lymphocytes [7]. *CD8A* mutations disrupt the expression of CD8^+^ T cells, impairing the immune system’s ability to clear intracellular pathogens [1,3,8].

The first individual with CD8α deficiency to be described in the literature presented with long-standing recurrent bacterial sinopulmonary infections, including documented incidences of *Haemophilus influenzae* and *Pseudomonas aeruginosa*, and subsequently developed severe, diffuse bronchiectasis. Despite supportive care, his pulmonary disease progressed to end-stage chronic respiratory failure, and he died at 33 years of age before lung transplantation could be performed [4,5]. Notably, two sisters in the same family were found to share the CD8α-deficient immunophenotype but remained clinically asymptomatic, emphasizing marked intrafamilial variability [5]. In a later report, a second symptomatic patient―an 18-year-old woman―experienced recurrent bronchiolitis, pneumonia, and otitis beginning in early infancy; over time, she developed left lower-lobe atelectasis (identified at around 4 years of age) and failure to thrive (recognized around 7 years of age) [4], further illustrating the broad spectrum of respiratory morbidity associated with CD8α deficiency. In the most recently published detailed clinical report prior to our case, the authors described a child with a *CD8A* missense variant and performed an extensive immunologic evaluation. That report highlighted that CD8α deficiency is associated with recurrent respiratory infections, but TCR-dependent T-cell proliferative responses remain largely preserved. Collectively, the findings broadened the recognized clinical spectrum of *CD8A*-related CD8 deficiency and supported the theory that affected individuals may present predominantly with sinopulmonary infectious complications in spite of otherwise relatively intact in vitro T-cell activation metrics [2].

In the current case, the identified homozygous nonsense variant (NM_001768.7:c.319C>T; p.Arg107Ter) creates a premature termination codon that is predicted to trigger nonsense-mediated mRNA decay, leading to markedly reduced/absent *CD8A* mRNA and loss of CD8α expression. The latter is expected to impair the CD8 coreceptor complex, as CD8α is a coreceptor component and is required for CD8β surface expression; in its absence, CD8β is retained and degraded [7,8]. This predicted loss-of-function mechanism is consistent with autosomal-recessive CD8 deficiency due to homozygous *CD8A* variants, which is typically characterized by absent CD8^+^ T cells with otherwise preserved lymphocyte subsets.

In this context, our patient’s results were consistent with prior reports describing early-onset sinopulmonary disease, characteristic immunophenotyping with absent CD8^+^ T cells, and variable degrees of chronic lung involvement. Importantly, our patient’s truncating p.Arg107Ter variant expands the mutational spectrum beyond previously described missense changes and further supports the marked phenotypic variability observed across affected individuals [2,4,5]. Because only a very small number of cases have been reported, drawing strong conclusions is difficult. However, the available literature shows that *CD8A* deficiency has a highly heterogeneous clinical phenotype, likely influenced by non-measurable factors such as environmental exposures to pathogens that rely on CD8-mediated clearance, as well as widespread vaccination programs that reduce respiratory infections and may slow the progression of lung disease. In addition, differences in how pneumonia, sinusitis, and bacterial bronchitis are recognized and managed across clinicians and centers can significantly affect disease progression even before the diagnosis is established.

Management of CD8α deficiency is primarily supportive and focuses on preventing recurrent respiratory infections that may lead to irreversible airway damage, particularly bronchiectasis. Because there is no evidence-based, disease-specific treatment, reported cases show variation in management across centers.

In our patient, therapy included optimizing airway clearance, tapering asthma medications, and initiating prophylactic azithromycin three times weekly. We did not initiate immunoglobulin replacement therapy because the patient’s immunoglobulin levels were normal, the degree of lung disease was very mild, and―as of now―there is insufficient evidence to support the routine use of this high-risk therapy, which carries potential for significant adverse effects. Notably, one of the previously reported cases required immunoglobulin therapy because the patient had advanced bronchiectasis, as outlined in Table 1 [2].

Overall, long-term care aims to preserve airway health and reduce infection-related morbidity in the absence of targeted therapy. Future multicenter, double-blinded randomized clinical trials are needed to determine the most effective treatment strategies for this rare condition.

This report has several limitations. First, as a single-patient observation, it cannot establish definitive genotype–phenotype correlations or explain the marked clinical variability described across affected individuals. Second, the functional consequences of p.Arg107Ter were inferred from its predicted loss-of-function effect (premature termination and likely nonsense-mediated decay) rather than confirmed through transcript/protein studies (e.g., *CD8A* mRNA quantification, Western blotting, or surface expression rescue assays) or comprehensive cytotoxic T-cell functional testing. Third, longer longitudinal follow-up is needed to better define the natural history, infection spectrum, and trajectory of chronic lung disease in this patient. Future research should prioritize systematic multicenter case collection/registries, standardized immunologic phenotyping (including antigen-specific cytotoxicity), and functional validation of truncating variants to clarify disease mechanisms, refine prognosis, and inform evidence-based approaches for the monitoring and management of this exceptionally rare primary immunodeficiency.

## 5. Conclusions

In this paper, we described a seven-year-old boy with CD8α deficiency due to a homozygous truncating *CD8A* variant (p.Arg107Ter), expanding the mutational spectrum of this exceptionally rare primary immunodeficiency. Clinically, his presentation highlights that *CD8A*/CD8α deficiency can resemble common pediatric respiratory disorders (e.g., asthma) yet be driven by an underlying T-cell subset defect, with characteristic immunophenotyping showing markedly reduced/absent CD8^+^ T cells and radiologic evidence of chronic sinopulmonary involvement. Together with prior reports demonstrating wide phenotypic variability—from asymptomatic individuals to progressive bronchiectasis and fatal respiratory failure—this case underscores the importance of early recognition and targeted immunologic evaluation in children with recurrent sinopulmonary infections to enable appropriate surveillance and preventive management.

## Figures and Tables

**Table 1:** Characteristics of the present case and those of CD8α deficiency (*CD8A* (OMIM: 608957) reported in the literature.

Reference	Country/Year	Patients (*n*)	Age (Y)/Gender (Male, Female)	Age of Onset	Race/Consanguinity	Complications	Genomic Position	DNA-Level Change	Protein/Mutation Type	Variant Classification	Outcome
Current patient	Saudi/2026	1	7/male	5 years	Middle Eastern/positive	Bronchiectasis/chronic sinusitis	2-86790412-G-A (GRCh38)	M_001768.7:c.319C>T	NP_001759.3:p.Arg107Ter; nonsense variant	Likely pathogenic	Alive
[5]	Spanish/2001	3 (index case was male with two sisters)	25/male	5 years	Gypsy/positive	Extensive bronchiectasis/chronic sinusitis	*CD8A* gene, exon 2 (immunoglobulin domain); cDNA position 331 (numbered from ATG start codon)	G→A at nucleotide 331 (HGVS-style: c.331G>A	Gly90→Ser (HGVS: p.Gly90Ser); missense variant	Disease-causing mutation	Deceased at age 33 years/respiratory failure. Two sisters healthy at time of report.
[4]	Spanish/2008	1	16/female	1 year	Gypsy/positive	Recurrent sinopulmonary infection and failure to thrive	*CD8A* gene, exon 2 (immunoglobulin domain); locus described in interval containing *CD8A/CD8B* on chromosome 2p32; mutation referenced as CD8A:c.331G>A	c.331G>A (G-to-A transition at nucleotide 331)	p.Gly111Ser; missense substitution	Pathogenic	Alive at time of report.
[2]	France/2015	1	14 years/male	3 months	Portuguese/positive	Bronchiectasis/chronic sinusitis	*CD8A* gene; homozygous variant in the immunoglobulin domain of CD8α; reported as c.331G>A	c.331G>A (homozygous)	p.Gly111Ser (G111S); missense variant	Pathogenic	Improved after IVIG replacement (0.5 g/kg every 3 weeks); respiratory function improved and daily amoxicillin stopped after 3 months; patient alive at publication.

## Data Availability

The original contributions presented in this study are included in the article. Further inquiries can be directed to the corresponding author.
